# A Catalytic Mechanism for Cysteine N-Terminal Nucleophile Hydrolases, as Revealed by Free Energy Simulations

**DOI:** 10.1371/journal.pone.0032397

**Published:** 2012-02-28

**Authors:** Alessio Lodola, Davide Branduardi, Marco De Vivo, Luigi Capoferri, Marco Mor, Daniele Piomelli, Andrea Cavalli

**Affiliations:** 1 Pharmaceutical Department, University of Parma, Parma, Italy; 2 Drug Discovery and Development, Italian Institute of Technology, Genova, Italy; 3 Department of Pharmacology, University of California Irvine, Irvine, California, United States of America; 4 Department of Pharmaceutical Sciences, University of Bologna, Bologna, Italy; University of Sydney, Australia

## Abstract

The N-terminal nucleophile (Ntn) hydrolases are a superfamily of enzymes specialized in the hydrolytic cleavage of amide bonds. Even though several members of this family are emerging as innovative drug targets for cancer, inflammation, and pain, the processes through which they catalyze amide hydrolysis remains poorly understood. In particular, the catalytic reactions of cysteine Ntn-hydrolases have never been investigated from a mechanistic point of view. In the present study, we used free energy simulations in the quantum mechanics/molecular mechanics framework to determine the reaction mechanism of amide hydrolysis catalyzed by the prototypical cysteine Ntn-hydrolase, conjugated bile acid hydrolase (CBAH). The computational analyses, which were confirmed in water and using different CBAH mutants, revealed the existence of a chair-like transition state, which might be one of the specific features of the catalytic cycle of Ntn-hydrolases. Our results offer new insights on Ntn-mediated hydrolysis and suggest possible strategies for the creation of therapeutically useful inhibitors.

## Introduction

N-terminal nucleophile (Ntn-) hydrolases are a superfamily of enzymes specialized in the cleavage of amide bonds [1]. Ntn-hydrolases become catalytically active after autocatalytic cleavage of an N-terminal peptide, which creates a novel N-terminal residue – usually a Ser, Thr, or Cys – that is responsible for amide bond cleavage [2]. As with classical amidases, Ntn-hydrolases are thought to catalyze the cleavage of their substrates by means of two consecutive reactions: (i) breakage of the amide bond with formation of an acylenzyme adduct; (ii) acylenzyme hydrolysis with regeneration of a catalytically competent enzyme.

Despite their common structural features, members of the Ntn-hydrolase family have evolved beyond any recognizable sequence homology [3]. They include structurally and functionally different enzymes, such as penicillin V acylase (PVA), used to produce semi-synthetic penicillins [4], and conjugated bile acid hydrolase (CBAH), which is responsible for the hydrolysis of bile salts and controls the balance of cholesterol into the enterohepatic circulation [5]. Ntn-hydrolase enzymes are also emerging as important targets for therapy. Notable examples include the subunit 20 S of the proteasome [6], which is inhibited by the anti-cancer drug bortezomib [7], N-acylethanolamine-hydrolyzing acid amidase (NAAA) [8], a potential target for anti-inflammatory and analgesic drugs [9], and acid ceramidase (AC) [10], a potential target for cancer chemosensitizing agents [11].

The crystal structure of CBAH from *Clostridium perfringens* was resolved [12] and recently used to build homology models of NAAA [9] and AC [10]. These models, which were validated by mutagenesis experiments [9], [10], suggest that CBAH, NAAA, and AC share a relatively well-conserved active site. In addition to the N-terminal catalytic cysteine (Cys2, according to the CBAH sequence), other conserved residues appear to be essential for the amidase activity of these enzymes [12]. These are Arg18 and Asp21, which undertake hydrogen bonds (H-bonds) with the sulfhydryl and alpha-amino groups of Cys2; Asn82 and Asn175, which form the putative oxyanion hole. Furthermore, kinetic experiments have shown that the hydrolytic activities of CBAH, NAAA, and AC display a bell-shaped pH dependency – with an optimal activity ranging from 4.5 for NAAA [13] and AC [14], to nearly 6 for CBAH [15]. These findings support the hypothesis that Ntn cysteine-hydrolases might have evolved to cleave their substrates using the same catalytic strategy.

**Figure 1:**
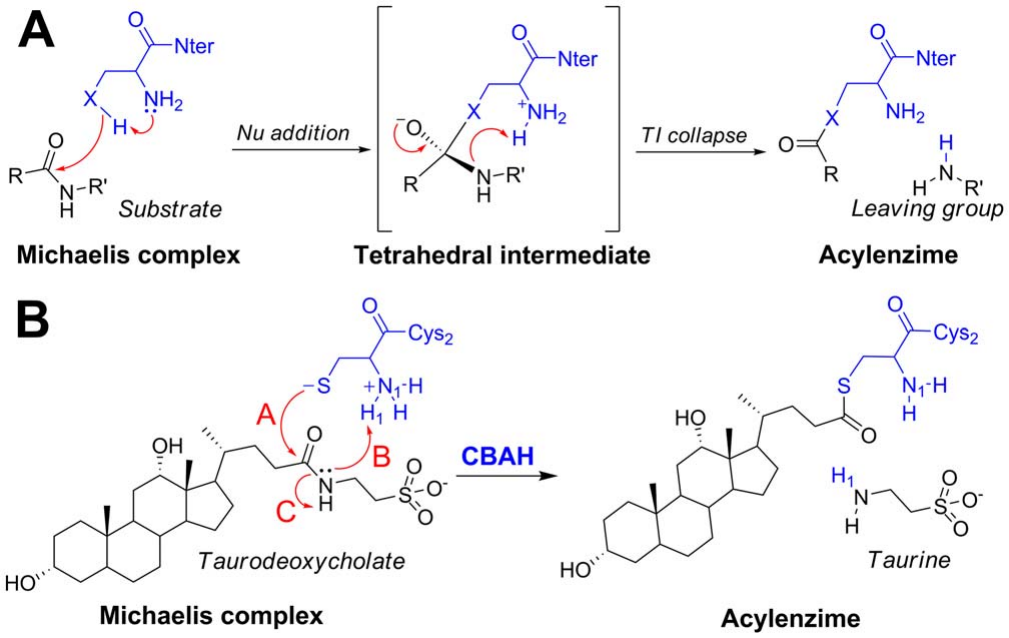
Catalytic mechanism of Ntn-hydrolases. Panel (A). The reaction begins when the nucleophilic oxygen/sulfur of Thr/Ser/Cys donates its proton to its own alpha-amino group and attacks the carbonyl carbon of the substrate [1], leading to a negatively charged tetrahedral intermediate (X represents oxygen or sulfur). The acylation step is completed when the alpha-amino group of the catalytic residue protonates the nitrogen of the scissile amide bond leading to the expulsion of the leaving group. Panel (B) First reaction of the catalytic mechanism of CBAH. **A**, **B**, and **C** are key steps for the cleavage of TAU amide bond.

A general mechanism for Ntn-hydrolases has been proposed by Oinonen and Rouvinen on the basis of structural data [1]. According to their hypothesis, the N-terminal catalytic residue of these enzymes lives in the neutral form. The reaction starts with a proton transfer between the nucleophile (OH or SH) of the catalytic residue and the alpha-amino group of the same amino acid (Figure 1A). Once deprotonated, the nucleophile attacks the carbonyl carbon of the substrate, leading to the formation of the tetrahedral intermediate (TI). The reaction becomes complete when the alpha-amino group of the catalytic residue donates a proton to the nitrogen of the scissile amide bond. In this way, an acylenzyme adduct is formed and the amino leaving group of the substrate is released (Figure 1A). This mechanism is consistent with current knowledge of serine [16], [17] and threonine Ntn-hydrolases [2], but is in contrast with experimental evidence on cysteine Ntn-hydrolases. For instance, since the optimal activity of many cysteine Ntn-hydrolases occurs at acidic pH [4], [8], [10], [15], it is unlikely that the alpha-amino group of the N-terminal Cys would act as a general base during the catalysis. In this respect, a recent study [18] has suggested that the key cysteine of an isopenicillin N-converting Ntn-hydrolase participates in the catalysis in its zwitterionic form, with the N-terminus positively charged and an anionic thiolate group.

Understanding the catalytic mechanism of cysteine Ntn-hydrolases would be important for three main reasons: (i) to define, at an atomic level, the mechanism of this reaction; (ii) to identify active-site residues important for catalysis; (iii) to provide insights for the rational design of pharmacologically useful inhibitors [19]. In the present study, to elucidate the catalytic mechanism of this class of enzymes, we investigated the first step of taurodeoxycholate (TAU) hydrolysis by CBAH (Figure 1B) using a hybrid quantum mechanics/molecular mechanics (QM/MM) technique [20], [21], a well-established approach in computational enzymology [22], [23]. Specifically, we calculated the free energy for TAU hydrolysis by using a conceptually innovative strategy based on enhanced sampling techniques, such as steered-molecular dynamics (steered-MD) and umbrella sampling (US) simulations, together with path collective variables (PCVs) [24]. This computational approach has been shown to be effective in studying reaction mechanisms and to explicitly account for anharmonicity and entropic effects [25]. Our analyses suggest that (i) the catalytic N-terminal cysteine participates in catalysis via its zwitterionic form; and (ii) the lowest free energy reaction pathway shows the formation of a zwitterionic tetrahedral adduct, in analogy with other cysteine hydrolases. Our calculations also reveal that the tetrahedral adduct is characterized by a cyclic “chair-like” structure, which might represent a significant signature of this class of enzyme.

## Results and Discussion

### Ionization state of Cys2

**Figure 2:**
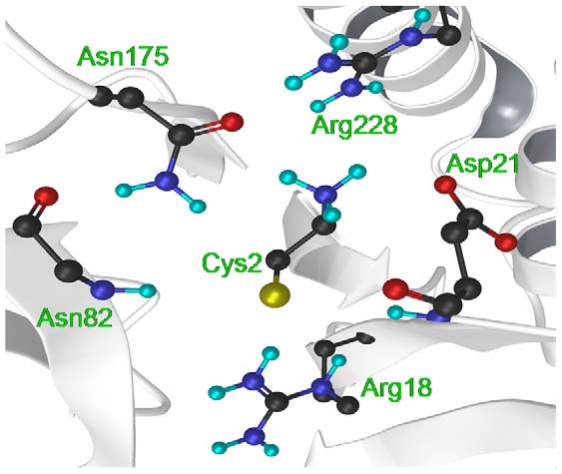
CBAH residues interacting with Cys2 as found in the crystal structure. Secondary structure elements of CBAH are displayed with gray cartoons, while carbon atoms are in black.

The determination of pKa values of the ionizable residues is at the basis for understanding the pH-dependent characteristics of proteins and catalytic mechanisms of many enzymes. The empirical PROPKA approach [26] was applied to calculate the pKa values of Cys2, starting from the crystallographic coordinate of CBAH from *Clostridium perfringens* [12]. The pKa values of Cys-NH_3_^+^ and Cys-SH groups were predicted to be ∼7.5 and ∼4, respectively. While the ionization state of the alpha-amino group of Cys2 (Cys-NH_3_^+^) was scarcely influenced by CBAH, when compared to solution (experimental pKa ∼8), the thiol group (Cys-SH) was significantly more acidic than in solution (experimental pKa of ∼9) [26]. Even taking into account the average error of PROPKA (26) in estimating the pKa of Cys-SH group (∼1 pKa unit), the observed pKa shift remained remarkably large (∼4–6 pKa units). A further analysis revealed that this shift was mainly due to electrostatic interactions between Cys2 sulfur atom and the conserved residues Arg18, Asp21, Asn82, and Asn175 (Figure 2, Table S1, and Table S2), suggesting that enzyme structure has evolved to stabilize the thiolate anion.

**Figure 3:**
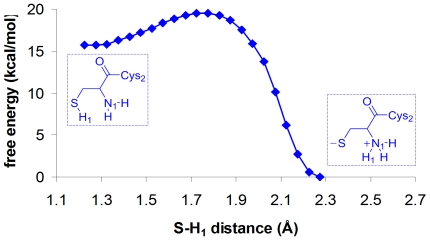
Free energy profile for Cys2 internal proton transfer. Free energy profile along S, H_1_ distance as obtained from umbrella sampling calculations and WHAM.

Since CBAH from *Clostridium perfringens* has an optimal hydrolytic activity in the pH interval 5.8–6.4 [15], it is likely that Cys2 participated to catalysis in zwitterionic form, i.e. Cys-S^−^/Cys-NH_3_^+^ ion pair (Figure S1). As the zwitterionic form of Cys2 was expected to live in equilibrium with its neutral form (Cys-SH/Cys-NH_2_), we calculated the potential of mean force for the intramolecular proton-transfer between the alpha-NH_3_^+^ and the S^−^ of Cys2 using umbrella sampling in combination with QM/MM calculations (see the Methods section). We clearly showed that the zwitterionic state of Cys2 was significantly more stable (∼15 kcal/mol, Figure 3) than the neutral one, indicating that the catalytically active state of Cys2 was Cys-S^−^/Cys-NH_3_^+^ ion pair. Similar findings have recently been reported for other cysteine proteases – i.e. cathepsine K [27] and papaine [28] –, where the catalytic machinery composed by a cysteine-histidine dyad was demonstrated to be catalytically active as a thiolate-imidazolium ion pair.

### Binding mode of TAU substrate within the CBAH active site

**Figure 4:**
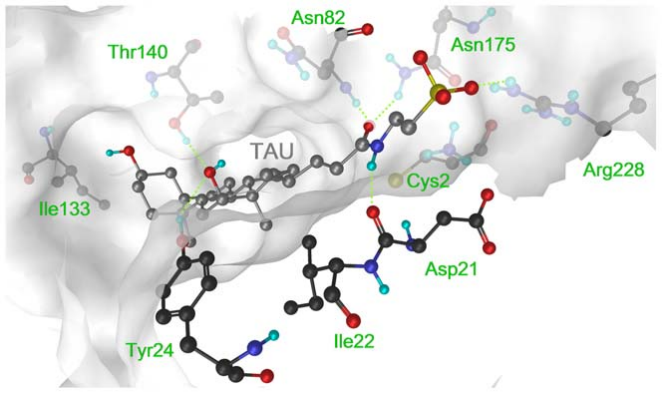
CBAH in its Michaelis complex with TAU. CBAH carbon atoms are in black, while TAU carbon atoms are in gray. The CBAH active site is represented as a solid van der Waals surface (white), which is occupied by TAU substrate. Relevant CBAH residues are displayed and labeled. Relevant H-bonds between the substrate and CBAH are shown as dotted green lines.

An important condition for investigating enzyme-catalyzed reactions is to build a reliable model system for the enzyme-substrate complex. This is not always easy because the so-called “Michaelis complex” structures are usually not available experimentally. Here, the availability of the crystal structure of CBAH from *Clostridium perfringens* in complex with its reaction products [12], namely deoxycholate and taurine, allowed us to easily detect the amino acids of the active site. Moreover, in agreement with the CBAH crystal structure [12], docking of TAU into the CBAH active site (Text S1) pointed mainly to one preferred binding mode, with the hydrophobic portion of TAU placed into the “cholyl” binding site [12], and the hydroxyl group in position 12 forming H-bonds with Tyr24 and Thr140 (Figure 4). The taurine moiety of TAU pointed toward a highly polar region composed of Asp21, Asn82, Asn175, and Arg228 of CBAH. While the sulfonate head formed a salt bridge with the guanidinium group of Arg228, the amide portion of TAU accepted H-bonds from the oxyanion hole residues Asn82 and Asn175, and donated an H-bond to Asp21 backbone oxygen (Figure 4). The identified CBAH-TAU complex underwent 20 ns of molecular dynamics (MD) calculations (see Methods). During MD simulations, TAU was remarkably stable thanks to two H-bonds with Asn82 and Asn175, and to a salt bridge between its sulfonate group and Arg228. These initial investigations showed that the CBAH-TAU Michaelis complex was suitable for subsequent studies on the CBAH reaction mechanism.

### Free energy simulations of the catalytic mechanism of CBAH

**Figure 5:**
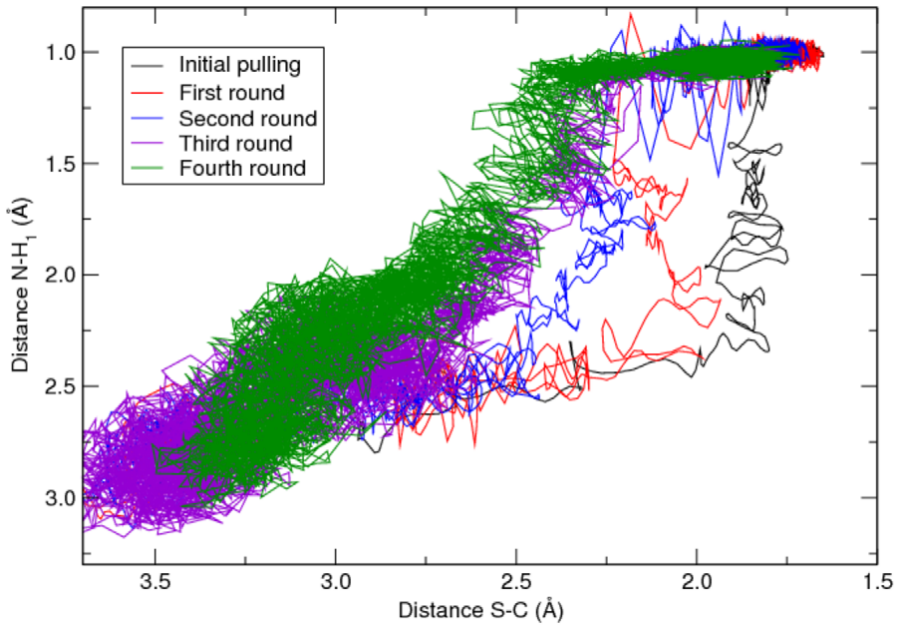
Evolution of the reaction path from steered-MD/PCVs simulations. The initial pulling showing the nucleophilic attack (described by the S, C distance), occurring before the proton transfer reaction (N, H_1_ distance), is displayed as a black line. The first steered-MD round is featured by a similar reaction path. Conversely, the last three steered-MD runs show a different mechanism, where protonation of the nitrogen (N) occurs at the same time (run three) or even before the nucleophilic attack. The steered-MD rounds third and fourth displayed a very similar distributions of the S-C and N-H_1_ distances, indicating the reaction path is converged in terms of explored configurations of the system.

Free energy simulations of multi-step enzymatic reactions are generally carried out by splitting the process into several consecutive events. While effective, this approach has two major limits: (i) it does not fully capture the concerted nature of the chemical process; (ii) the alignment of the free energy profiles of each step can lead to relevant approximations. The path collective variables scheme overcomes these limits [25], as it describes multi-step reactions with only two variables (*S* and *Z*) that represent the progression along (*S*) and the distance from (*Z*) a reactive guess path (see Methods for further details). In the present work, we generated a guess reaction path using steered-MD, assuming a mechanism similar to that proposed by Oinonen and Rouvinen [1], i.e. with the nucleophilic addition followed by the TI collapse (Figure 1A). In particular, the reaction was initially divided into three consecutive steps (**A**, **B**, **C** in Figure 1B; see Methods for further details). The path was then iteratively optimized by means of steered-MD simulations carried out in the *S* and *Z* space, and on the converged path obtained by steered-MD (Figure 5), umbrella sampling was carried out to estimate the free energy associated with the catalytic process.

**Figure 6:**
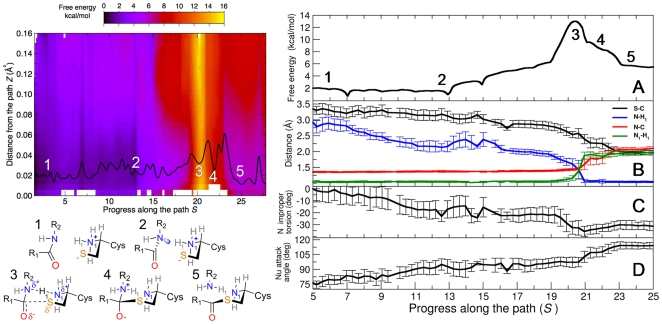
Free energy surface (FES) for TAU hydrolysis catalyzed by CBAH. Left panel. Bi-dimensional FES in the *S* and *Z* space from US simulations. The minimum free energy path is displayed with a continuous black line. Configurations **1**–**5** are crucial structures identified along the reaction path, *S*. Right panel, subpanel (A). Projection of the FES of TAU hydrolysis on *S*. Right panel, subpanel B. Relevant interatomic distances (reported as average over US with error bars representing the standard deviations) are plotted as function of *S*. Right panel, subpanel C. Improper torsion of the amide nitrogen (N) of TAU as function of *S*. Right panel, subpanel D. Nucleophile attacking angle as function of *S*.

The minimum free energy path (Figure 6) resulted in a region of low *Z* values, indicating that the optimized steered-MD path was reliable. Based on the free energy profile of Figure 4, the catalytic mechanism can be divided into 5 chemically relevant regions: (i) the Michaelis complex (i.e. the reactants; region **1**); (ii) a distorted Michaelis complex featuring a pyramidalized TAU nitrogen (region **2**); (iii) the transition state (TS), dominated by protonation of TAU nitrogen (region **3**); (iv) the tetrahedral adduct (TA) which did not correspond to a well-defined free energy minimum (region **4**); and (v) the acylenzyme intermediate (region **5**). The free energy surface (FES) of Figure 6 also shows that, while the profile was almost flat in regions **1** and **2**, a significant increment in the free energy was observed approaching region **3** (the TS area), which was characterized by a saddle-like surface. Region **4** accounted for the nucleophilic attack and evolved to **5**, the acylenzyme adduct, spontaneously.

In Figure 6, right panel, the mono-dimensional projection of the FES and the evolution of relevant interatomic distances are plotted as functions of the *S* collective variable (progress along the path). Between regions **1** and **2** (*S* between 1 and 13), a geometrical distortion of the Michaelis complex occurred, as a consequence of the interaction between Cys2 and the amide group of TAU. In particular, the thiolate group of Cys2 approached the carbonyl carbon (S-C distance from ∼3.3 Å in **1** to ∼3.0 Å in **2**), while H_1_ moved towards TAU nitrogen (N-H_1_ distance from ∼3.0 Å in **1** to ∼2.1 Å in **2**). Moreover, the TAU nitrogen underwent a pyramidalization process (i.e. its improper torsion changed from ∼0° in **1** to ∼−16° in **2**), which was facilitated by the formation of an H-bond with Asp21 backbone carbonyl. The nucleophile attacking angle (S-C-O) increased from ∼75° in **1** to a more favorable value of ∼100° in **2**. Despite these geometrical changes, the free energy profile was quite flat between **1** and **2**. In the *S* interval 13–19, the free energy increased of ∼6 kcal/mol due to a further shortening of the S-C and N-H_1_ distances. N-H_1_ distance was found to be the pivotal variable for the reaction to occur. This was confirmed by the gradient analysis reported in Figure S2, which shows the key structural components of the mean force, passing from the reactants to the products (25). After *S* = 19, the free energy associated to the catalysis reached the value of 13.5±0.2 kcal/mol (Text S2). This corresponded to the activation free energy (**3**, the TS, see Figure 6). Remarkably, this value is in very good agreement with that obtained experimentally, corresponding to ∼15.0 kcal/mol [29].

The gradient analysis (Text S3 and Figure S2) shows that the change of N_1_-H_1_ and N-H_1_ distances played a major role in the TS crossing (*S* between 19 and 21). At the TS, the N-H_1_ and N_1_-H_1_ distances were of ∼1.4 Å and ∼1.3 Å, respectively. The H_1_ transfer from NH_3_^+^ of Cys2 to the amide nitrogen of TAU (N_1_) was therefore identified as the key chemical event for CBAH-catalyzed hydrolysis.

**Figure 7:**
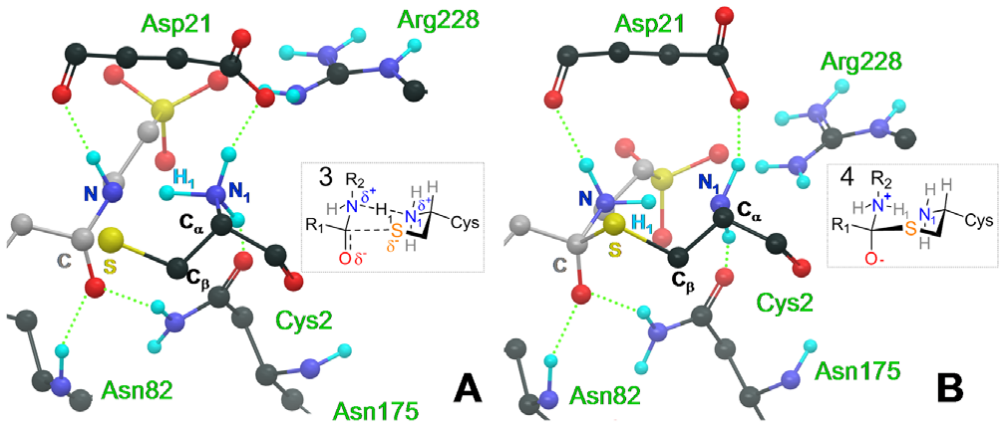
Transition state (TS) and tetrahedral adduct (TA) geometries identified along the path. Left panel (A). TS structure of TAU (gray carbons) hydrolysis catalyzed by CBAH (black carbons). H_1_ is nearly equidistant between N and N_1_ favoring the formation of a pseudo chair structure. Right panel (B) Zwitterionic TI. In both panels, H-bonds are shown as dotted green lines, while secondary structure elements of CBAH are omitted for clarity.

As shown in Figure 7A, the TS assumed a “chair-like” structure, mainly due to the alignment of N-H_1_-N_1_ atoms, and to the position of the TAU carbonyl with respect to the Cys sulfur atom (S-C distance = ∼2.6 Å and S-C-O angle = ∼100°). This chair-like geometry was stabilized by a complex network of H-bonds (Figure 7A). The TAU carbonyl oxygen occupied a pseudo-axial position forming two H-bonds, with Asn82 and Asn175. Similarly, the TAU amide hydrogen formed an H-bond with the Asp21 backbone oxygen. The Cys2 alpha-amino group also formed two H-bonds, with Asp21 and Asn175. Remarkably, a chair-like structure has previously been observed when simulating the reaction mechanism of a threonine Ntn-hydrolase, with a small model system [30]. Therefore, we here suggest that a chair-like structure of the TS might be an important feature for the Ntn-hydrolase family of enzymes. The proton transfer between N_1_ and N was completed at *S* = 21. However, the free energy of the system was still relatively high, as the nucleophilic attack carried out by Cys2 on TAU was still happening. This event was completed at *S* = 22, where a zwitterionic TA (**4** in Figure 6) was finally obtained (S-C distance of ∼2.2 Å and S-C-O angle of ∼105°). The chair-like conformation was conserved in the TA (Figure 7B), which was well stabilized by the CBAH active site residues. In particular, the negatively charged oxygen of TAU was stabilized by H-bonds with Asn82 and Asn175 of the oxyanion hole, while the Cys2 alpha-amino group H-bonded Asp21 and Asn175. In addition, the leaving group nitrogen formed two H-bonds with the Asp21 backbone carbonyl and with the neutral Cys2 N-terminus (N_1_). The pseudo-axial and pseudo-equatorial hydrogens of N_1_ established H-bonds with Asn175 and Asp21, respectively (Figure 7B). The TA was ∼4 kcal/mol more stable than the TS (Figure 6). Although leaving group protonation and nucleophilic attack appeared to be consecutive events, the free energy profile (Figure 6) indicated that these two steps were concerted, similarly to what has been reported for amide hydrolysis catalyzed by classical cysteine hydrolases, such as papaine [31] or cathepsin K [27].

It is worth mentioning that the reaction mechanism described in Figure 6 is remarkably different from the step-wise mechanism proposed by Oinonen and Rouvinen (Figure 1A), which reported the presence of a stable tetrahedral intermediate [1]. This further highlights the need to employ path-based rather than step-wise approaches for studying complex biochemical reaction mechanisms [32].

Starting from the metastable tetrahedral adduct, at *S* = 24 the N-C bond was completely broken, with the two atoms being ∼2.1 Å far apart. This barrier-less event led to the formation of the acylenzyme, **5**. An sp^3^ to sp^2^ hybridization change of the carbonyl carbon of TAU occurred (Figure 6, right panel, plot D), and a thioester species was formed at the CBAH active site. Consistently with this event, the S-C-O angle was ∼118°. The expulsion of the leaving group (i.e. taurine) was facilitated by Arg228, which formed a salt bridge with the sulfonate group of taurine. The acylenzyme was ∼6 kcal/mol less stable than the reactants. This result is consistent with the transient character of the acylenzyme, which has to be cleaved by an incoming water molecule to restore a catalytically competent form of the enzyme.

### Steered-MD simulations of TAU hydrolysis in CBAH and in aqueous solution

The mechanism of TAU hydrolysis catalyzed by CBAH was also investigated by analyzing the work curve obtained from the final steered-MD simulation performed in the S/Z space (Text S4). Although the steered-MD-derived activation energy was ∼18 kcal/mol, ∼5 kcal/mol higher than that calculated with US, the steered-MD work profile was remarkably similar to the US free energy profile (Figure S3), suggesting that our steered-MD protocol can be applied to qualitatively analyze the energetics of reaction pathways. We therefore studied the reaction between a free zwitterionic cysteine and TAU in aqueous solution by steered-MD simulations (Text S5 and Figure S4). This represented a “reference reaction” [33] and its analysis could help in understanding the catalytic role of CBAH, regardless of alternative acylation mechanisms (i.e. involving a different protonation state of the cysteine) in aqueous solution. Therefore, this should be considered a computational experiment that had only the scope to address the role of the protein environment on the reaction, and it was not meant to describe realistically this mechanism in water. Here too, the reaction was initially divided into three consecutive steps (**A**, **B**, **C** in Figure S5) and then, a steered-MD/PCV protocol was applied to identify the optimal path in the *S*/*Z* space (Text S5).

Comparison of the steered-MD-derived work profiles showed that the two reaction mechanisms (i.e. in CBAH and in water) were remarkably similar, with protonation of the leaving group concerted with the nucleophilic attack (Figure S6). In aqueous solution too, the TS showed a chair-like structure similar to that identified for CBAH-catalyzed reaction (Figure S7). As expected, the barrier calculated from work profile in water (∼25.0 kcal/mol) was ∼7 kcal/mol higher than that observed in CBAH. Indeed, polar residues of CBAH active site are well-organized to electrostatically stabilize the TS (Figure 7A), whereas in solution, water molecules have to pay an energetic penalty to reorganize their dipoles toward the TS distribution charge [33].

### Effects of mutations on CBAH catalysis

Sequence analysis [12], [29] and mutagenesis data on the CBAH homologue NAAA [9] have pointed out that Arg18, Asp21, Asn82, Asn175, and Arg228 are crucial for catalysis. Therefore, the role of these conserved amino acids on the CBAH catalysis was investigated by simulating the first step of TAU hydrolysis in the presence of “zero point charge” CBAH mutants (Text S6), by applying the steered-MD/PCVs protocol. The zero point charge mutation has already been used to clarify the role of active site residues in enzyme catalysis [34].

**Figure 8:**
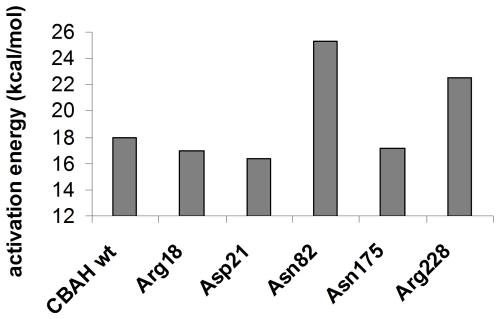
Activation barriers for TAU hydrolysis as obtained from steered-MD/PCVs simulations. Barriers are estimated from work profiles and are refereed to CBAH wild type (wt) and to zero-point charge mutants.

The steered-MD-derived work profiles of the catalysis using different mutants resembled that of the wild-type enzyme. However, each mutant had a different effect on the activation barrier for TAU hydrolysis, as calculated from the work profile (Figure 8). Zero point charge mutation of Arg18, Asp21, and Asn175 had a small effect on the barrier height (less than 2 kcal/mol), suggesting that their role is to control the protonation state of Cys2 (see above). Conversely, mutation of Asn82 and Arg228 led to a relevant increase of the activation barrier. This was ∼6 kcal/mol more than the wild type for Asn82 zero point charge mutant, indicating that the oxyanion hole plays a crucial role in TS stabilization. Mutation of Arg228 increased the barrier of ∼4 kcal/mol, suggesting that the electrostatic interaction between the sulfonate group of TAU and the guanidinium moiety of Arg228 can be fundamental for an efficient leaving group protonation and expulsion. These results are in good agreement with experimental data available for Ntn-hydrolase mutants, thus supporting the reliability of the simulations described above.

In summary, in this study we used an innovative computational approach based on steered-MD, umbrella sampling, and the path collective variable approach to characterize, in the QM/MM framework, the first reaction in the catalytic cycle of CBAH, a prototypical cysteine Ntn-hydrolase. The activation free energy calculated for this catalysis was in excellent agreement with experimental data. Moreover, our studies revealed a novel mechanism in which protonation of the leaving group is elegantly concerted with the nucleophilic attack. The reaction path was characterized by a chair-like TS structure, stabilized by the catalytic pocket of CBAH. Several residues involved in the stabilization of the TS are highly conserved among cysteine Ntn-hydrolases, suggesting that the CBAH active site is pre-organized to accommodate a cyclic TS structure, and to ultimately catalyze the reaction. Another characteristic feature of the reaction mechanism proposed here is the presence of a zwitterionic tetrahedral adduct, as a cross-road for acylenzyme formation. Finally, a computational mutagenesis analysis indicated that Asn82 and Arg228 have a direct role in TS stabilization. Mutation of Arg18 and Asp21 had little or no effect on the barrier, confirming that the primary role of these residues is to modulate the pKa of Cys2, which was catalytically competent in its zwitterionic form. The highly conserved Asn175 had a minor role in TS stabilization, as this residue was intimately engaged in recognizing the substrate through the H-bond formation in the Michaelis complex. The mechanism described here provides new insights into the catalytic mechanism of cysteine Ntn-hydrolases, which might help to understand the biochemical functions of these enzymes, and to design novel inhibitors with potential therapeutic application [35].

## Methods

### Model building

The CBAH-TAU Michaelis complex was built starting from the publicly available crystal structure of CBAH in complex with taurine and deoxycholate (PDB ID code 2bjf) [12], and applying a standard docking protocol (Text S1). The best docking complex was selected and immersed in a box of TIP3P water molecules [36] and neutralized with 8 Na^+^ ions by using the xleap tool. The total system size amounted to 51,858 atoms (box size of 70.3 Å×80.6 Å×92.1 Å). The system was minimized and then thermalized (300 K) and pressurized (1 atm) in the NPT ensemble (i.e. constant temperature and pressure) for 20 ns of molecular dynamics (MD) simulations, using the AMBER99SB force field [37], in the sander module of AMBER10 [38]. Long-range electrostatics was treated using the particle mesh Ewald with 128×128×128 grid points. The covalent bonds involving hydrogen atoms were constrained with the SHAKE [39] algorithm allowing a time-step of 2 fs.

### The QM/MM scheme

The equilibrated Michaelis complex was further thermalized (300 K) in the NVT ensemble for 20 ps using the QM/MM scheme implemented in the sander module of the AMBER10 suite [38]. Cys2 and the 2-pentanamido ethansulfonic acid fragment of TAU were treated with the self-consistent charge-density functional tight-binding (SCC-DFTB) approach [40], as implemented in sander [41], [42]. All the other atoms were described with AMBER99SB force field [37]. The resulting QM system was composed of 40 atoms including two link atoms. One link atom was placed along the C-C bond connecting the 2-pentanamido ethansulfonic chain of TAU to its steroidal scaffold, while the other was placed along the C-N bond connecting Cys2 with Thr3. The *adjust_q* function of AMBER was applied to conserve the total charge of the system [38]. Additionally, we also verified that the charge on the alpha carbon of Thr3 (0.011 e-) was similar to that observed when the whole amide group of Cys2 was included in the QM selection (0.007 e-).

During QM/MM MD simulations (either plain or biased), all the atoms of the system (including hydrogens) were free to move, and a time step of 0.2 fs was used to integrate the equation of motion. Although SCC-DFTB method has some limitations, it has become increasingly widely applied to biological problems [40], and it has been reported to provide results in agreement with DFT calculations [43], [44]. Furthermore, its efficiency made it feasible to sample millions of conformations for enzyme systems and to determine the free energy profiles of the enzyme-catalyzed reactions [45], complementing results obtained with *ab initio* free energy methods [46], [47].

### Internal proton transfer in Cys2

An umbrella sampling calculation was performed by imposing constraining potentials along the S, H_1_ distance (Figure 3), with a force constant of 300 kcal/mol Å^−2^. In this way, the proton H_1_ was gradually moved from the alpha-NH_3_^+^ group of Cys2 to the –S^−^ group of the same cysteine. The spacing between the harmonic potentials was 0.05 Å for a total of twenty-two simulation windows. The statistics for each harmonic potential was acquired by starting the simulation from the final configuration of the previous biased simulation. The sampling for each harmonic potential consisted of 40 ps. We discarded the statistics from the first 20 ps, to allow a suitable relaxation from the previous imposed bias. The final free energy profile was obtained by using the weighted histogram analysis method (WHAM) [48] on the collected statistics, thus allowing the estimate of the free energy difference between zwitterionic and neutral state of Cys2.

### Definition of the reaction path

A preliminary path between reactants and products was generated using steered-MD and simple reaction coordinates (RCs). The reaction was initially divided into three consecutive steps (**A**, **B**, **C** in Figure 1B). In **A**, the nucleophilic attack of CBAH thiolate on the carbonyl carbon of TAU was simulated using S-C distance as RC. This step led to the formation of a tetrahedral intermediate (TI). In **B**, TI protonation was simulated using N-H_1_ distance as RC. Finally in **C**, the expulsion of the leaving group was simulated using C-N_1_ distance as RC. In all these steps, the RCs were sequentially pulled to their ideal target values by using a force constant of 300 kcal/mol Å^−2^ for a total simulation time of 10 ps. From the resulting steered-MD trajectory, a set of frames, representing the preliminary path between reactants and products, were extracted. The C(O)NH TAU atoms and S, C_beta_, C_alpha_, N_1_, and H_1_ atoms of Cys2 atoms were used as template structure for the frame selection procedure for PCVs (23). We obtained 30 equally spaced frames (average root mean square deviation of 0.09 Å) that were used to define the collective variables *S* and *Z* in a subsequent steered-MD simulation. In detail, *S* and *Z* describe the position of a point in the configurational space *(R)* relative to an initial path, and are defined as follows:
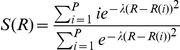


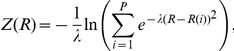
where *i* is a discrete index (in this study ranging from 1 to 30), and *(R−R(i))^2^* is calculated as the mean square displacement from the initial path.

The system was thus pulled along *S* from 1 (reactants) to 30 (products), applying a force constant of 300 kcal/mol at a velocity of 0.5 *S* units per ps (Figure 5, black line). The variable *Z* was constrained by a quartic wall to allow a relative freedom of the system to relax, while preventing it to escape from the reactive region. The upper limit over *Z* was set to 0.005 Å^2^ with a force constant of 200 kcal/mol Å^−8^.

At the end of the steered-MD/PCVs simulation, a new set of frames was extracted and employed as a novel reference path for a subsequent steered-MD/PCVs run. This procedure was repeated iteratively until the reaction path did not change passing from a steered-MD/PCVs run to a new one (see Figure 5). Finally, PCVs were employed in US simulations, using the converged steered-MD configurations as a reference path. US simulations were carried out using a spring constant of 1 kcal/mol and 2,500 kcal/mol Å^−4^ on *S* and *Z*, respectively. 4 ps of simulation per umbrella were performed for a total of 2.7 ns. The final free energy surface was obtained by using WHAM approach, with an average error of 0.2 kcal/mol (Text S2). All the steered-MD and US simulations were carried out with sander module of AMBER10 patched with the PLUMED package [49].

## Supporting Information

Figure S1 **Effect of pH on CBAH activity.** The figure is adapted from Gopal-Srivastava R, Hylemon PB (1988) Purification and characterization of bile salt hydrolase from *Clostridium perfringens*. *J Lipid Res* 29: 1079–1085.
(TIF)

Figure S2 **Projection of the gradients of** ***S*** **.** Absolute value of the projection of the gradients of S over the gradient of relevant interatomic distances (see Text S3 for details). High values denote interatomic distances which play a pivotal role determining the mean force along the reaction progress.
(TIF)

Figure S3 **Work profile of the first step of TAU hydrolysis by CBAH over** ***S*** **.** Work profile of the first step of TAU hydrolysis by CBAH over *S* by steered-MD, compared with free energy profile obtained with umbrella sampling (and also reported in Figure 6A of the main text).
(TIF)

Figure S4 **Reactant complex formed by Cys-OMe and TAU substrate.** Reactant complex formed by Cys-OMe and TAU substrate as a model system to simulate amide hydrolysis in solution. Water molecules within 6 Å from the reactive center are displayed.
(TIF)

Figure S5 **First step of TAU hydrolysis by Cys-OMe in solution.** **A**, **B**, and **C** are key steps for the cleavage of TAU amide bond.
(TIF)

Figure S6 **Comparison between TAU hydrolysis in CBAH and in solution.** Activation barriers for the first step of TAU hydrolysis catalyzed by CBAH (line) and in aqueous solution (dashed line), estimated from work profiles obtained from steered-MD/PCVs simulations. In the lower panel, relevant distances are plotted as a function of *S* (progress along the path).
(TIF)

Figure S7 **Superposition of the transition state (TS) structures for the reaction in CBAH solution and in CBAH.** TS structures were identified along steered-MD/PCVs simulations. The TS geometry for the reaction in aqueous solution is reported with white carbon, while the TS geometry for the enzyme catalyzed reaction is reported with black carbons.
(TIF)

Table S1 **pKa prediction of titrable sites in CBAH by PROPKA.**
(DOC)

Table S2 **PROPKA contributions to the final pKa for Cys2 thiol group.**
(DOC)

Text S1 **CBAH-TAU model building.**
(DOC)

Text S2 **Convergence of free energy obtained from US/PCVs simulations.**
(DOC)

Text S3 **Gradient Analysis.**
(DOC)

Text S4 **Free energy for TAU hydrolysis in CBAH by steered-MD/PCVs.**
(DOC)

Text S5 **Free energy for TAU hydrolysis in solution by steered-MD/PCVs.**
(DOC)

Text S6 **Zero point charge mutants of CBAH.**
(DOC)
