# Supplementary figures and images for: A Catalytic Mechanism for Cysteine N-Terminal Nucleophile Hydrolases, as Revealed by Free Energy Simulations
Source: PLoS One. 2012 Feb 28;7(2):e32397. doi: 10.1371/journal.pone.0032397 (PMC3289653; doi:10.1371/journal.pone.0032397)

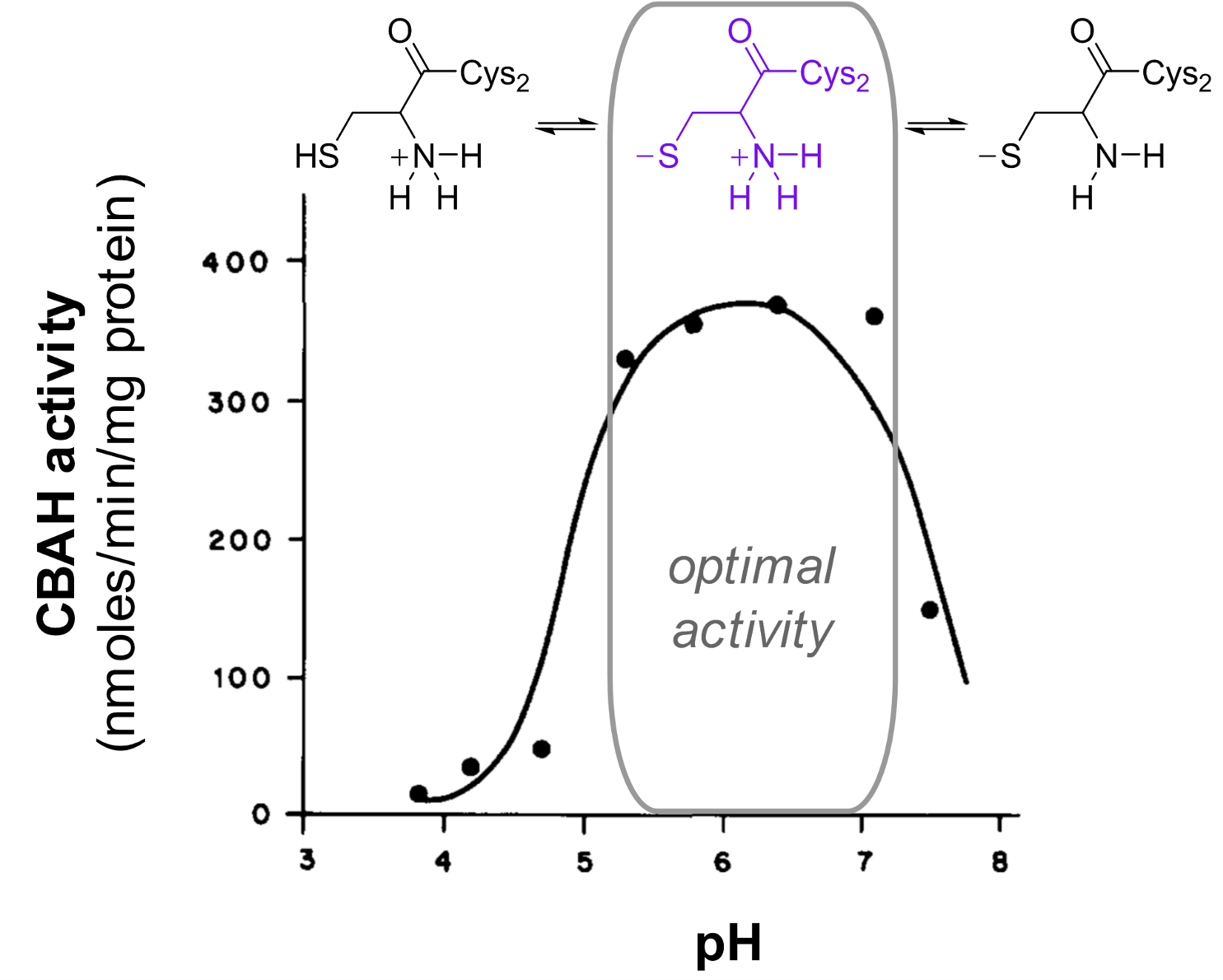

Supplement: Figure S1 — Effect of pH on CBAH activity. The figure is adapted from Gopal-Srivastava R, Hylemon PB (1988) Purification and characterization of bile salt hydrolase from Clostridium perfringens. J Lipid Res 29: 1079–1085. (TIF) [file pone.0032397.s001.tif]

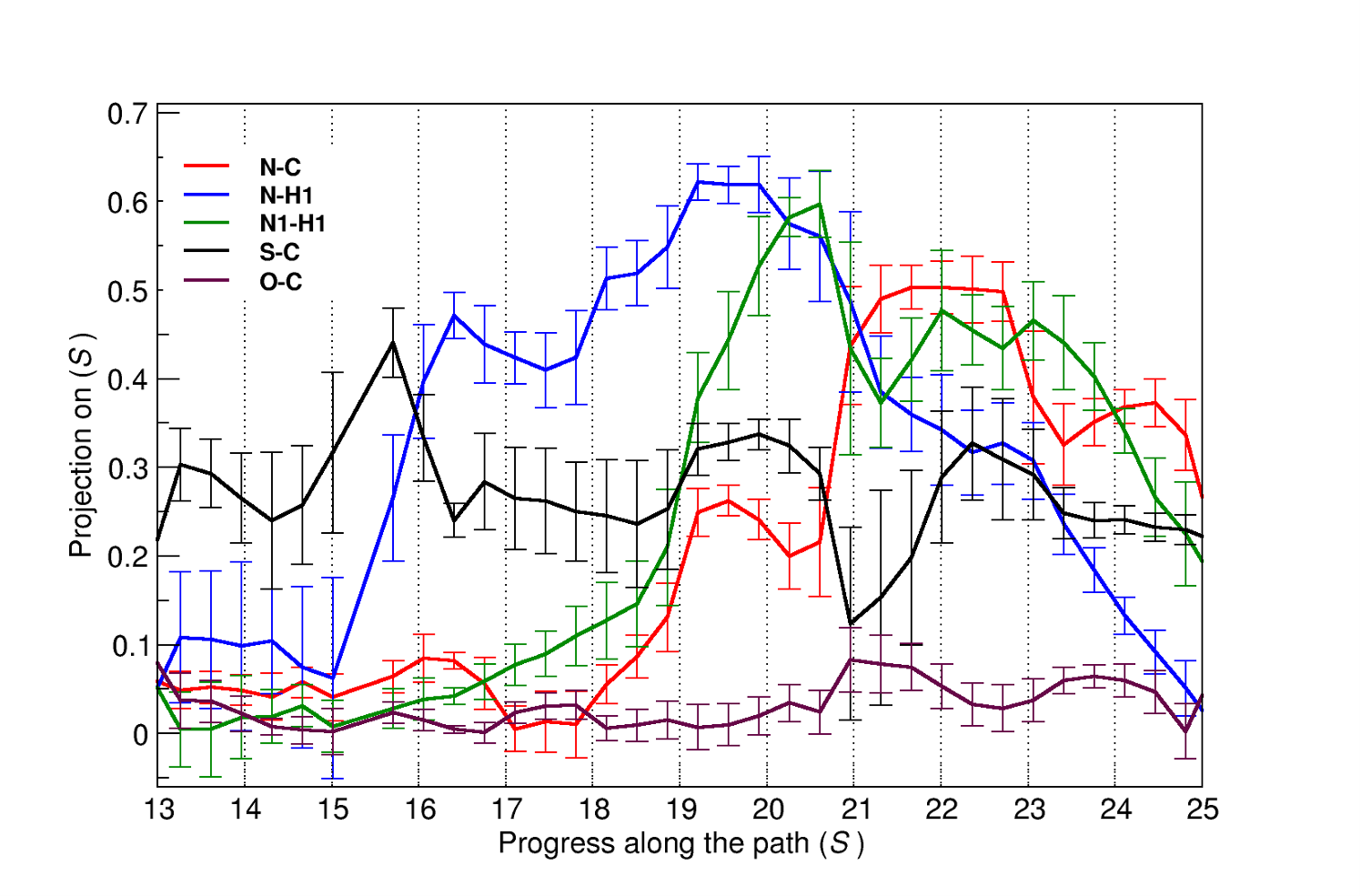

Supplement: Figure S2 — Projection of the gradients of S . Absolute value of the projection of the gradients of S over the gradient of relevant interatomic distances (see Text S3 for details). High values denote interatomic distances which play a pivotal role determining the mean force along the reaction progress. (TIF) [file pone.0032397.s002.tif]

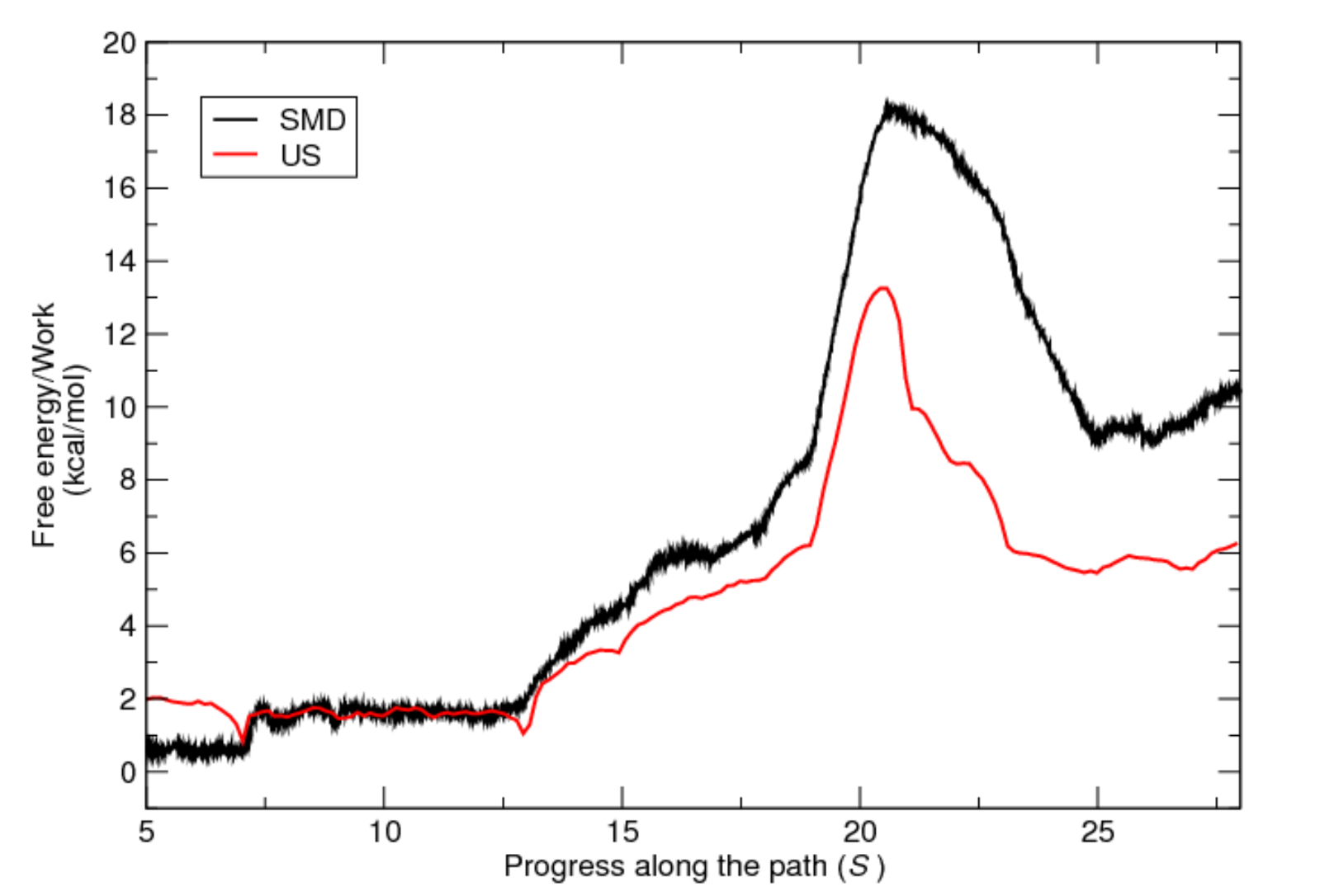

Supplement: Figure S3 — Work profile of the first step of TAU hydrolysis by CBAH over S . Work profile of the first step of TAU hydrolysis by CBAH over S by steered-MD, compared with free energy profile obtained with umbrella sampling (and also reported in Figure 6A of the main text). (TIF) [file pone.0032397.s003.tif]

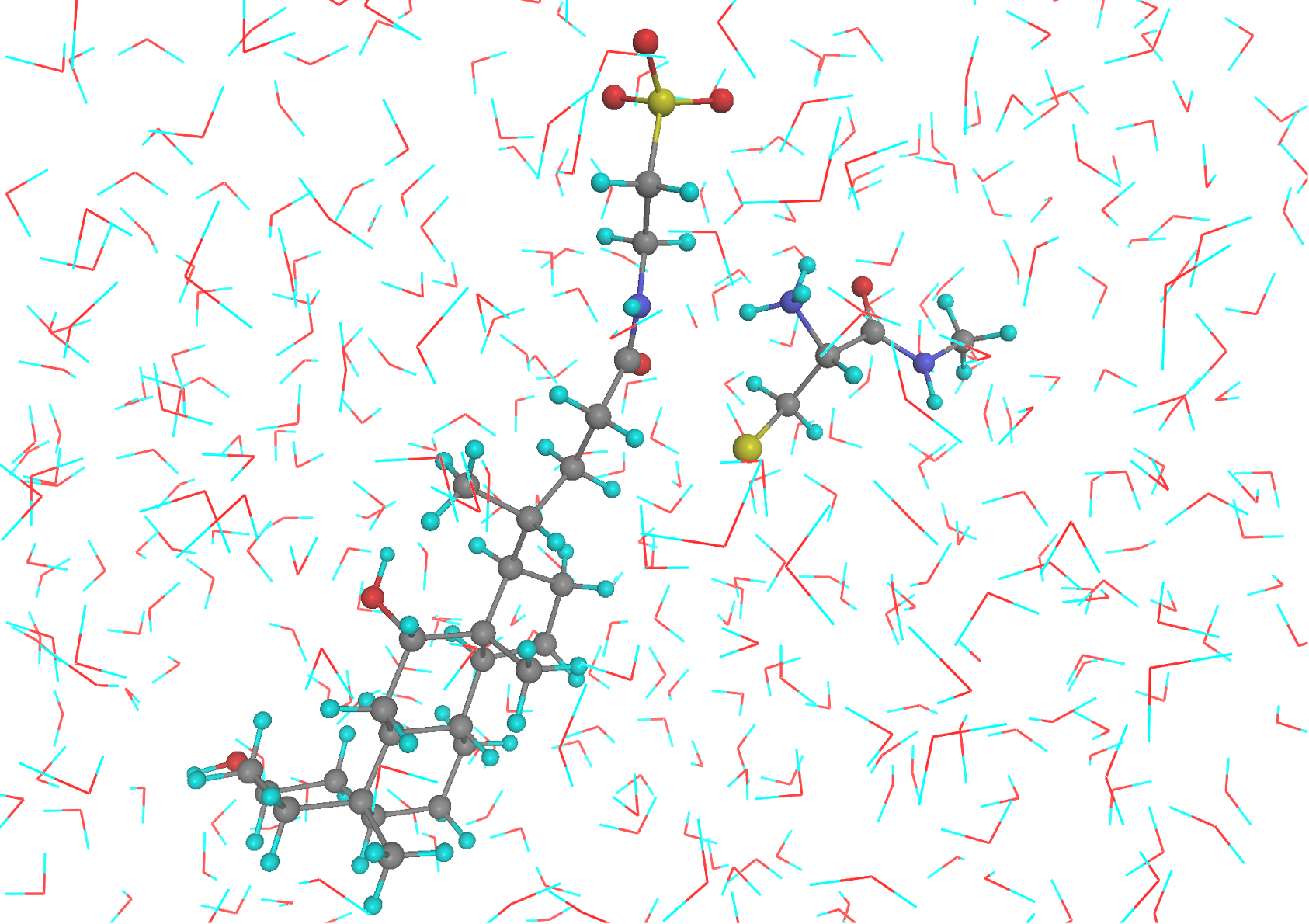

Supplement: Figure S4 — Reactant complex formed by Cys-OMe and TAU substrate. Reactant complex formed by Cys-OMe and TAU substrate as a model system to simulate amide hydrolysis in solution. Water molecules within 6 Å from the reactive center are displayed. (TIF) [file pone.0032397.s004.tif]

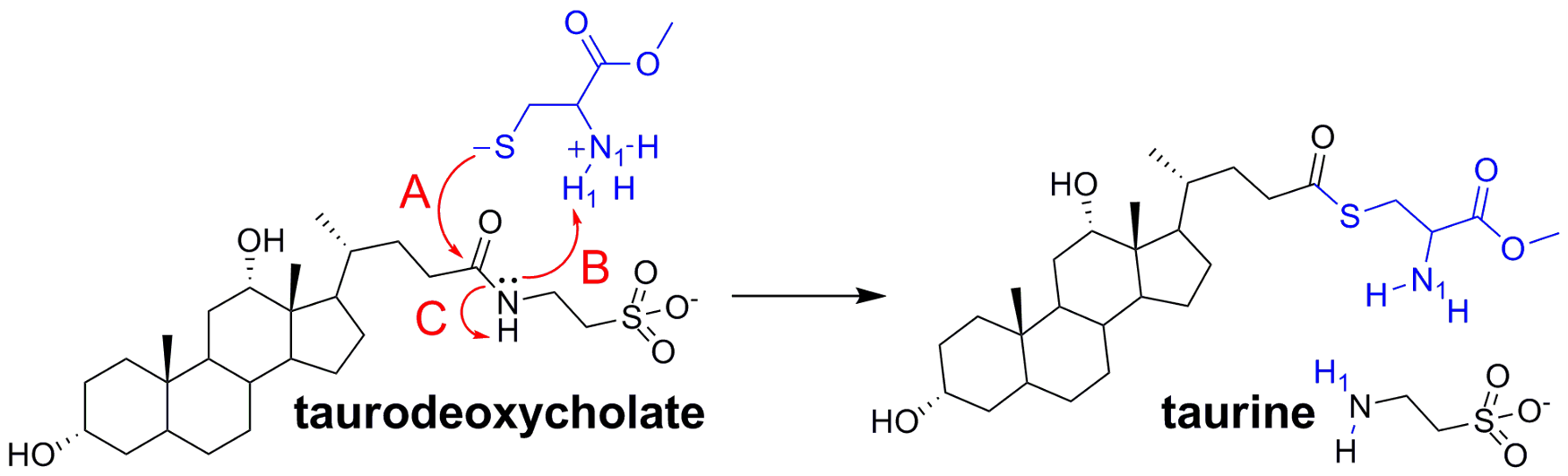

Supplement: Figure S5 — First step of TAU hydrolysis by Cys-OMe in solution. A, B, and C are key steps for the cleavage of TAU amide bond. (TIF) [file pone.0032397.s005.tif]

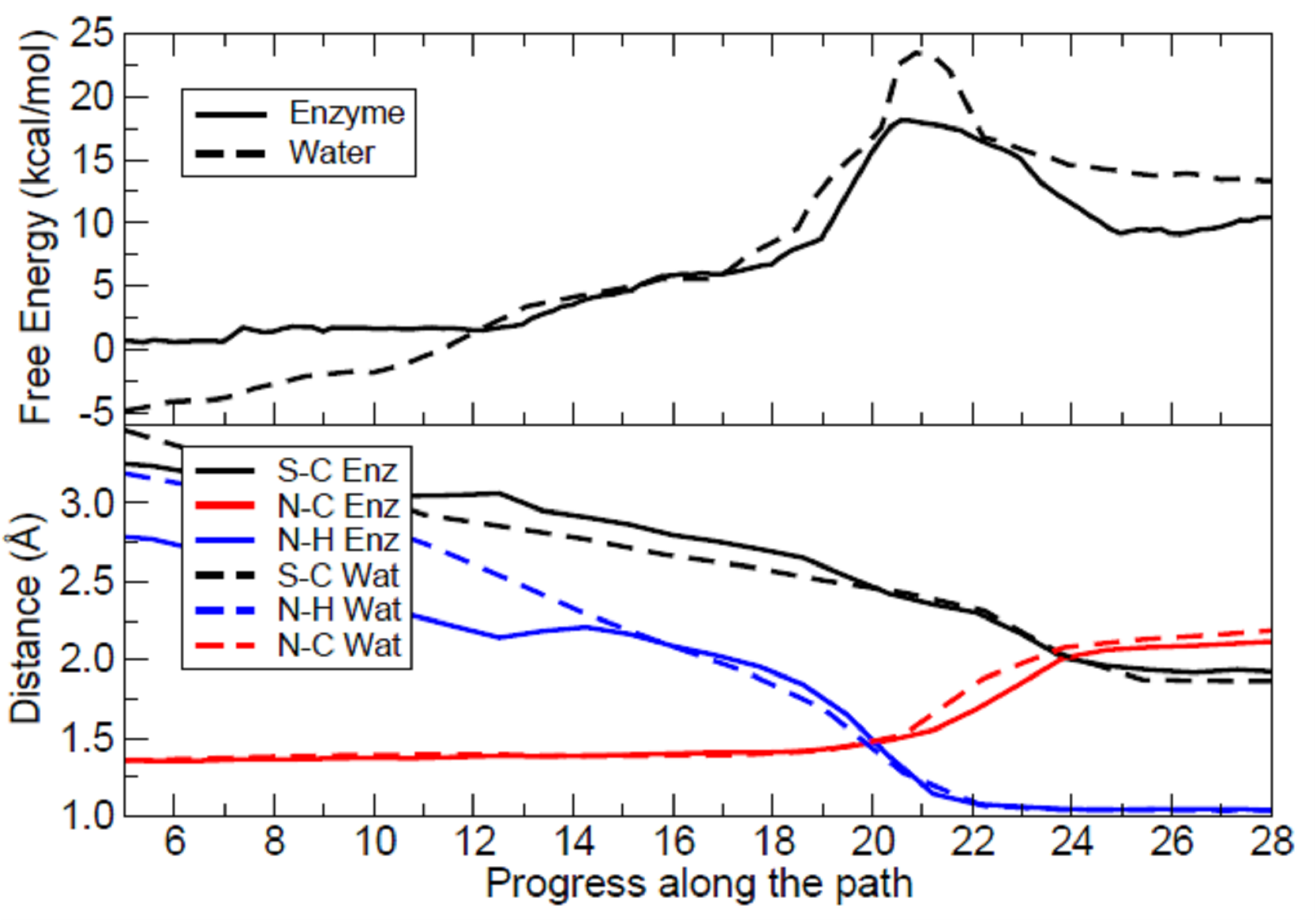

Supplement: Figure S6 — Comparison between TAU hydrolysis in CBAH and in solution. Activation barriers for the first step of TAU hydrolysis catalyzed by CBAH (line) and in aqueous solution (dashed line), estimated from work profiles obtained from steered-MD/PCVs simulations. In the lower panel, relevant distances are plotted as a function of S (progress along the path). (TIF) [file pone.0032397.s006.tif]

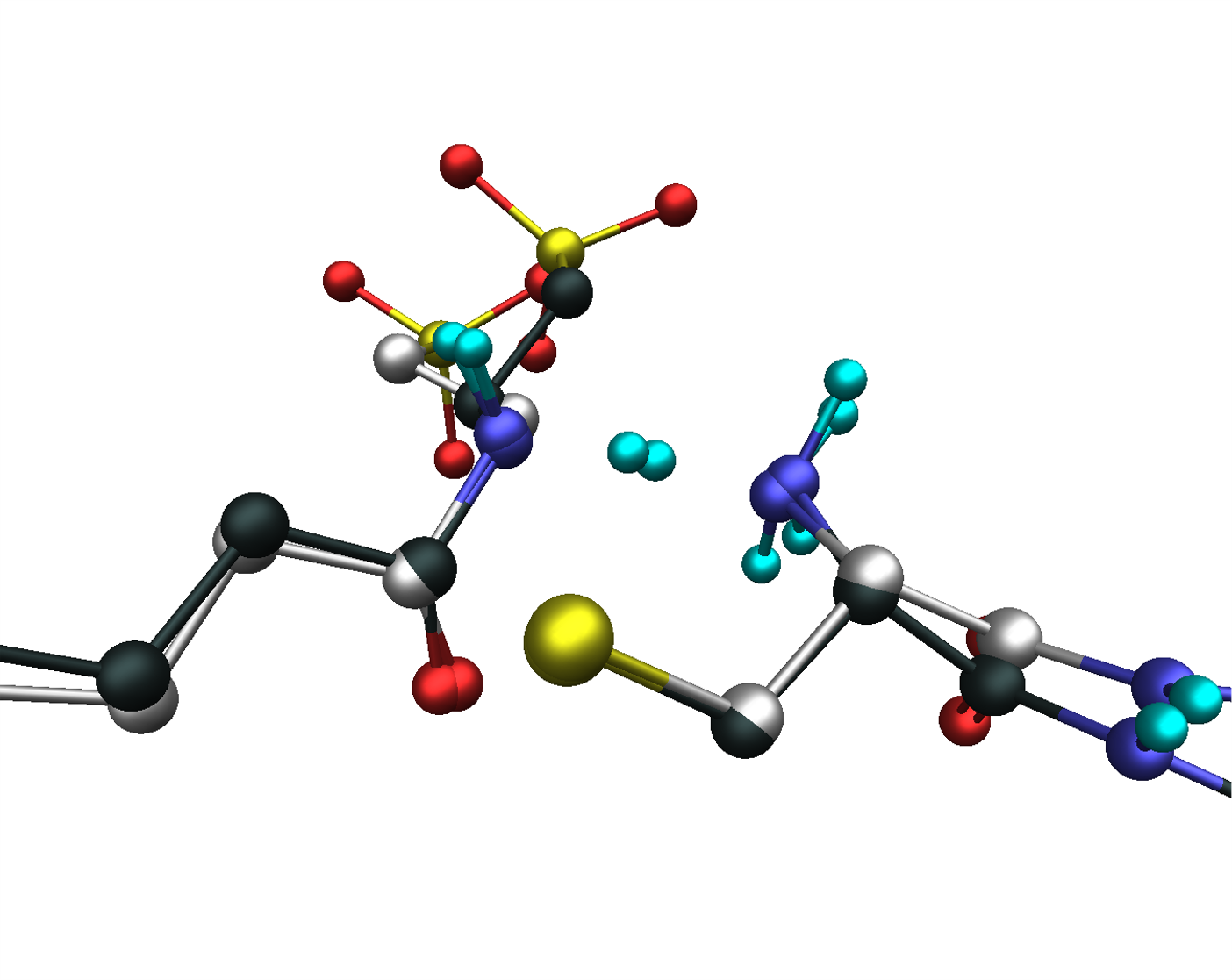

Supplement: Figure S7 — Superposition of the transition state (TS) structures for the reaction in CBAH solution and in CBAH. TS structures were identified along steered-MD/PCVs simulations. The TS geometry for the reaction in aqueous solution is reported with white carbon, while the TS geometry for the enzyme catalyzed reaction is reported with black carbons. (TIF) [file pone.0032397.s007.tif]
