# Supplementary material for: A Catalytic Mechanism for Cysteine N-Terminal Nucleophile Hydrolases, as Revealed by Free Energy Simulations
Source: PLoS One. 2012 Feb 28;7(2):e32397. doi: 10.1371/journal.pone.0032397 (PMC3289653; doi:10.1371/journal.pone.0032397)
Supplement: Table S1 — pKa prediction of titrable sites in CBAH by PROPKA. (DOC) [file pone.0032397.s008.doc]

**Table S1. pKa prediction of titrable sites in CBAH by PROPKA [[[1]](#endnote-2)].**

| Residue | Predicted pKa  in CBAH | Experimental pKa  in solution | Residue | Predicted pKa  in CBAH | Experimental pKa  in solution |
| --- | --- | --- | --- | --- | --- |
| ASP11 | 3.80 | 3.80 | **CYS2** | **4.20** | **9.00** |
| ASP21 | 4.66 | 3.80 | CYS39 | 10.19 | 9.00 |
| ASP62 | 3.37 | 3.80 | CYS78 | 12.88 | 9.00 |
| ASP63 | 3.87 | 3.80 | CYS281 | 11.28 | 9.00 |
| ASP69 | 3.57 | 3.80 | CYS283 | 7.62 | 9.00 |
| ASP93 | 4.08 | 3.80 | TYR24 | 11.46 | 10.00 |
| ASP130 | 2.67 | 3.80 | TYR52 | 11.49 | 10.00 |
| ASP148 | 1.52 | 3.80 | TYR64 | 13.89 | 10.00 |
| ASP167 | 3.14 | 3.80 | TYR86 | 10.00 | 10.00 |
| ASP180 | 2.51 | 3.80 | TYR103 | 9.81 | 10.00 |
| ASP204 | 3.82 | 3.80 | TYR189 | 10.00 | 10.00 |
| ASP222 | 0.86 | 3.80 | TYR194 | 13.41 | 10.00 |
| ASP237 | 2.76 | 3.80 | TYR278 | 14.66 | 10.00 |
| ASP243 | 4.08 | 3.80 | TYR289 | 17.26 | 10.00 |
| ASP245 | 3.87 | 3.80 | TYR290 | 10.92 | 10.00 |
| ASP248 | 3.04 | 3.80 | TYR291 | 9.79 | 10.00 |
| ASP274 | 5.53 | 3.80 | **Nterm (Cys2)** | **7.58** | **8.00** |
| ASP303 | 3.50 | 3.80 | LYS38 | 10.43 | 10.50 |
| ASP310 | 3.31 | 3.80 | LYS42 | 10.43 | 10.50 |
| GLU8 | 4.64 | 4.50 | LYS45 | 10.50 | 10.50 |
| GLU23 | 3.73 | 4.50 | LYS46 | 10.43 | 10.50 |
| GLU47 | 4.64 | 4.50 | LYS51 | 10.36 | 10.50 |
| GLU73 | 1.62 | 4.50 | LYS74 | 10.50 | 10.50 |
| GLU92 | 4.77 | 4.50 | LYS91 | 10.43 | 10.50 |
| GLU95 | 4.61 | 4.50 | LYS97 | 10.43 | 10.50 |
| GLU117 | 4.57 | 4.50 | LYS120 | 10.29 | 10.50 |
| GLU118 | 3.10 | 4.50 | LYS124 | 10.29 | 10.50 |
| GLU121 | 4.12 | 4.50 | LYS152 | 10.50 | 10.50 |
| GLU135 | 4.50 | 4.50 | LYS160 | 10.50 | 10.50 |
| GLU157 | 2.24 | 4.50 | LYS162 | 10.43 | 10.50 |
| GLU161 | 4.64 | 4.50 | LYS201 | 10.29 | 10.50 |
| GLU199 | 4.50 | 4.50 | LYS241 | 10.22 | 10.50 |
| GLU251 | 4.64 | 4.50 | LYS244 | 10.50 | 10.50 |
| GLU270 | 3.79 | 4.50 | LYS272 | 10.29 | 10.50 |
| GLU271 | 4.50 | 4.50 | LYS286 | 10.36 | 10.50 |
| GLU285 | -0.22 | 4.50 | LYS306 | 10.50 | 10.50 |
| GLU295 | 3.42 | 4.50 | LYS315 | 10.50 | 10.50 |
| GLU307 | 3.91 | 4.50 | LYS318 | 10.50 | 10.50 |
| GLU313 | 4.57 | 4.50 | LYS321 | 10.36 | 10.50 |
| Cterm | 3.27 | 3.20 | ARG18 | 13.14 | 12.50 |
| HIS14 | 7.86 | 6.50 | ARG35 | 11.66 | 12.50 |
| HIS143 | 5.10 | 6.50 | ARG193 | 12.29 | 12.50 |
| HIS182 | 2.71 | 6.50 | ARG228 | 10.32 | 12.50 |
| HIS254 | 6.50 | 6.50 | ARG231 | 10.89 | 12.50 |
| HIS327 | 6.43 | 6.50 | ARG236 | 10.28 | 12.50 |
| TYR294 | 8.92 | 10.00 | ARG263 | 12.08 | 12.50 |
| TYR317 | 13.27 | 10.00 | ARG267 | 11.80 | 12.50 |
| TYR319 | 11.91 | 10.00 |  |  |  |

1. . Li H, Robertson AD, Jensen, JH (2005) Very fast empirical prediction and rationalization of protein pKa values. *Proteins*, 61:704-721. [↑](#endnote-ref-2)
