# Supplementary material for: A Catalytic Mechanism for Cysteine N-Terminal Nucleophile Hydrolases, as Revealed by Free Energy Simulations
Source: PLoS One. 2012 Feb 28;7(2):e32397. doi: 10.1371/journal.pone.0032397 (PMC3289653; doi:10.1371/journal.pone.0032397)
Supplement: Table S2 — PROPKA contributions to the final pKa for Cys2 thiol group. (DOC) [file pone.0032397.s009.doc]

**Table S2. PROPKA contributions to the final pKa for Cys2 thiol group.**

| Residue | Predicted pKa  in CBAH | Experimental pKa  in solution | Desolvation | Local  Effects | Sidechain  Hydrogen bond | Backbone  Hydrogen bond | Coulombic  Interaction |
| --- | --- | --- | --- | --- | --- | --- | --- |
| CYS2 | 4.20 | 9.00 | 2.80 | 0.07 | -0.69 ASN175 | -2.35 ASP21 | 1.34 ASP21 |
|  |  |  |  |  | -1.60 ARG18 | -2.36 ASN82 | -2.01 ARG18 |
