# Supplementary material for: A Catalytic Mechanism for Cysteine N-Terminal Nucleophile Hydrolases, as Revealed by Free Energy Simulations
Source: PLoS One. 2012 Feb 28;7(2):e32397. doi: 10.1371/journal.pone.0032397 (PMC3289653; doi:10.1371/journal.pone.0032397)
Supplement: Text S1 — CBAH-TAU model building. (DOC) [file pone.0032397.s010.doc]

## Text S1. CBAH-TAU model building

The CBAH-TAU Michaelis complex was built using the publicly available crystal structure of CBAH in complex with taurine and deoxycholate [[[1]](#endnote-2)]. Non-protein atoms (taurine and deoxycholate) were deleted from the active site, while hydrogen atoms were added to CBAH structure using Maestro program [[[2]](#endnote-3)]. Protonation state of titrable groups was assigned with PROPKA as implemented in VEGA [[[3]](#endnote-4)]; taking into account that CBAH has optimal catalytic activity at pH 6.

TAU substrate was placed into CBAH active site using Glide 5.0 [[[4]](#endnote-5)] starting from minimum-energy conformations of TAU, placed in an arbitrary starting position within a region centred on residues Cys2, Asp21 and Asn175, using enclosing and bounding boxes of 20 Å and 10 Å on each side, respectively. Van der Waals radii of the protein atoms were not scaled, while van der Waals radii of the ligand atoms, having partial atomic charges between -0.15 and 0.15, were scaled by a factor of 0.8. Standard Precision (SP) mode was applied and docking solutions were ranked according to the G_score_ value.

1. . Rossocha M, Schultz-Heienbrok R, von Moeller H, Coleman JP, Saenger W (2005) Conjugated bile acid hydrolase is a tetrameric N-terminal thiol hydrolase with specific recognition of its cholyl but not of its tauryl product. Biochemistry 44: 5739-5748. [↑](#endnote-ref-2)
2. . Maestro, version 8.5; Schrodinger, LLC: New York, 2008 [↑](#endnote-ref-3)
3. . Pedretti A, Villa L, Vistoli G (2004) VEGA: an open platform to develop chemo-bio-informatics applications, using plug-in architecture and script programming. J Comput Aided Mol Des. 18: 167-173. [↑](#endnote-ref-4)
4. . Friesner RA, Banks JL, Murphy RB, Halgren TA, Klicic JJ, Mainz DT, Repasky MP, Knoll EH, Shelley M, Perry JK, Shaw DE, Francis P, Shenkin PS (2004) Glide: a new approach for rapid, accurate docking and scoring. 1. Method and assessment of docking accuracy. J Med Chem 47: 1739-1749. [↑](#endnote-ref-5)
