# Supplementary material for: A Catalytic Mechanism for Cysteine N-Terminal Nucleophile Hydrolases, as Revealed by Free Energy Simulations
Source: PLoS One. 2012 Feb 28;7(2):e32397. doi: 10.1371/journal.pone.0032397 (PMC3289653; doi:10.1371/journal.pone.0032397)
Supplement: Text S2 — Convergence of free energy obtained from US/PCVs simulations. (DOC) [file pone.0032397.s011.doc]

# Text S2. Convergence of free energy obtained from US/PCVs simulations

Error estimate of the free energy calculation was calculated by taking half the statistics from each of the biased calculations of US and repeating WHAM procedure for the two generated statistics. Each statistics was acquired within 2 ps of MD simulations in 400 different umbrellas in S and Z space. The two free energies obtained were aligned to their respective average. The global error was calculated as the difference in free energy between the two surfaces, divided by two. The resulting average error on the final surface was therefore estimated to be 0.2 kcal/mol.
