# Supplementary material for: A Catalytic Mechanism for Cysteine N-Terminal Nucleophile Hydrolases, as Revealed by Free Energy Simulations
Source: PLoS One. 2012 Feb 28;7(2):e32397. doi: 10.1371/journal.pone.0032397 (PMC3289653; doi:10.1371/journal.pone.0032397)
Supplement: Text S3 — Gradient Analysis. (DOC) [file pone.0032397.s012.doc]

# Text S3. Gradient Analysis

Path collective variables provide an effective reference system to map concerted motion and create an adaptive space to perform enhanced sampling calculations. While this approach, similarly to other path methods, provides reliable and reproducible free energy surfaces, it suffers from difficulties in its interpretation. In particular, since PCVs enclose many effects, it is useful to decompose the contribution of each degree of freedom so to detect a-posteriori the real determinants that allow the reaction to occur along a low free energy pathway.

In each biased simulation, the force on one atom is exerted through the gradient of the CV by using the following chain rule:

$$F_{x}(s(R))=-\frac{\partial U_{bias}(s(R))}{\partial s(R)}\frac{\partial s(R)}{\partial x}$$

where $F_{x}(s(R))$ is the force on the coordinate of one atom $x$ from the bias potential, $U_{bias}(s(R))$ is the bias potential which is exerted through the order parameter $s(R)$ that depends on the coordinate of all the atoms $R$. While the derivative respect $s(R)$ is identical for all the atom involved in defining the CV, the derivative respect $x$ is not. Therefore those atoms that present a high $\frac{\partial s(R)}{\partial x}$ value are expected to largely contribute to the progress of the CV.

Extending further this concept, by projecting the gradient of a PCV over the gradient of another variable, suitably normalized, one would get the degree of similarity of the force exerted by PCV to the force that would be exerted by a different descriptor (e.g. a distance, a torsion, etc.). In particular, the projection of $s(R)$ on a variable $CV(R)$ along a trajectory may be defined as:

$$P_{CV}(s_{0})=\left\langle\frac{\left|\nabla s(R)\cdot\nabla CV(R)\right|}{\left\|\nabla s(R)\right\|\left\|\nabla CV(R)\right\|}\delta(s(R)-s_{0})\right\rangle$$

where the average is taken over the all trajectory or snapshots available.

This quantity can be useful in rationalizing the different contributions that may come from a curvilinear path dealing with concerted motion of a certain number of atoms.

For the specific case of TAU hydrolysis in CBAH, we calculated the projection along *S* path variable on various distances involved in the transition state and we found that the increase of free energy was mostly associated with the tightening of N-H_1_ distance while the transition state was stongly associated with the N_1_-H_1_ bond breaking (Figure S2). Further decrease of free energy was provided by nucleophile attack that reduces N-C distance.

#
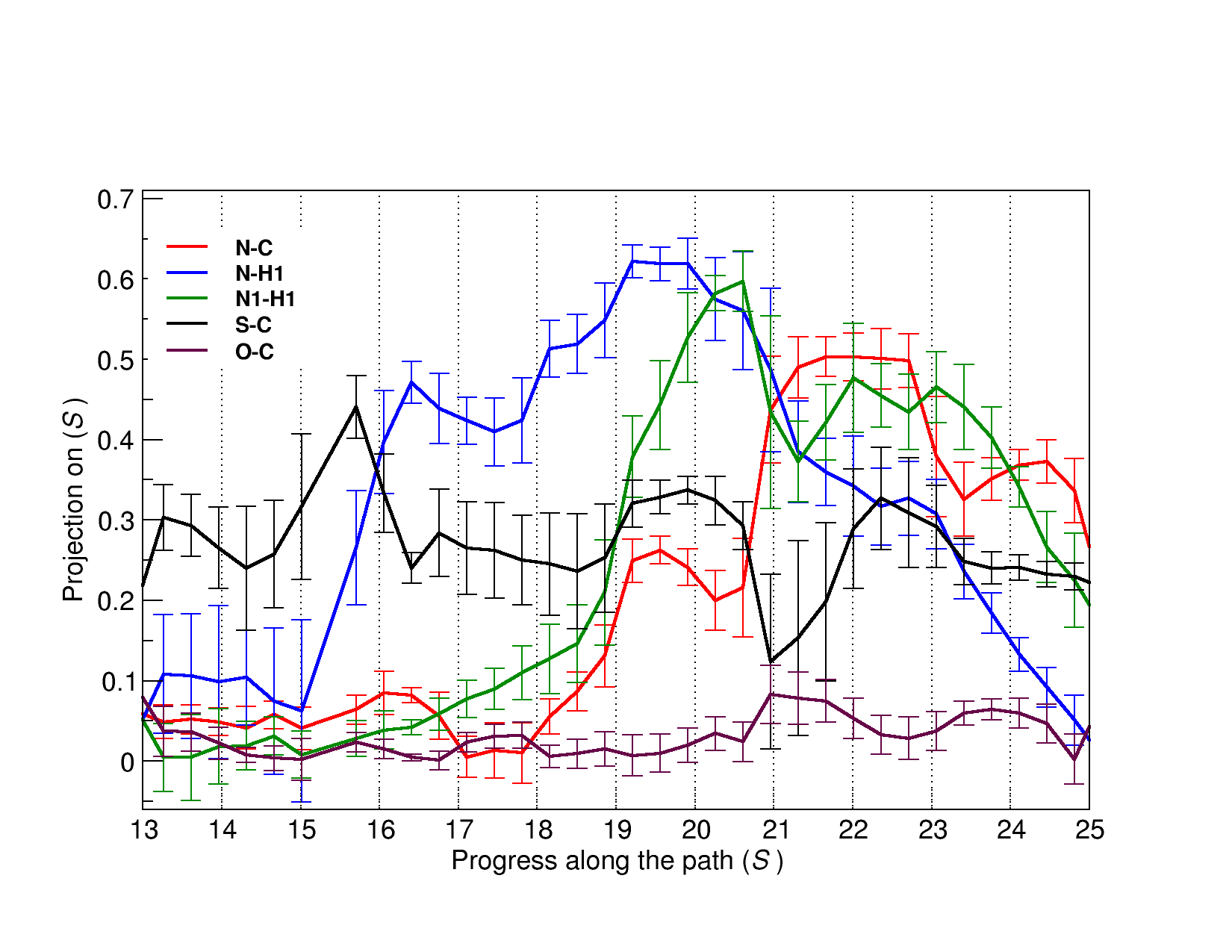


**Figure S2.** **Projection of the gradients of *S***

Absolute value of the projection of the gradients of S over the gradient of relevant interatomic distances (see above). High values denote interatomic distances which play a pivotal role determining the mean force along the reaction progress.
