# Supplementary material for: A Catalytic Mechanism for Cysteine N-Terminal Nucleophile Hydrolases, as Revealed by Free Energy Simulations
Source: PLoS One. 2012 Feb 28;7(2):e32397. doi: 10.1371/journal.pone.0032397 (PMC3289653; doi:10.1371/journal.pone.0032397)
Supplement: Text S4 — Free energy for TAU hydrolysis in CBAH by steered-MD/PCVs. (DOC) [file pone.0032397.s013.doc]

# Text S4. Free energy for TAU hydrolysis in CBAH by steered-MD/PCVs

Here we compare the minimum free energy path obtained via US (see Figure S3, red line) with the work profile obtained via steered-MD simulations (Figure S3, black line). The steered-MD simulations were carried out by steering along the path variable S with a spring constant of 300 kcal/mol at a velocity of 0.5 S units per ps (for a total simulation time of ~50 ps). As expected, the calculated work was higher than the free energy calculated via US (which was calculated with a total simulation time of 2.7 ns) due to the contribution of dissipative work. Nevertheless, it is worth noting that the shape of the work-curve was remarkably similar to the free energy profile obtained with US simulations. This justified the use of steered-MD to compare the reaction in aqueous solution with that in the enzyme (see below).


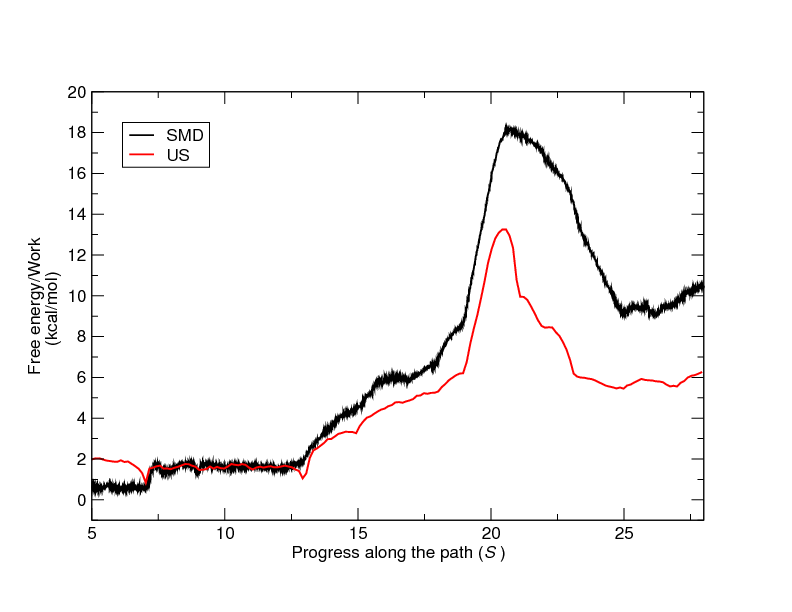


**Figure S3.** **Work profile of the first step of TAU hydrolysis by CBAH over *S***

Work profile of the first step of TAU hydrolysis by CBAH over *S* by steered-MD, compared with free energy profile obtained with US (and also reported in Figure 4A of the main text).
