# Supplementary material for: A Catalytic Mechanism for Cysteine N-Terminal Nucleophile Hydrolases, as Revealed by Free Energy Simulations
Source: PLoS One. 2012 Feb 28;7(2):e32397. doi: 10.1371/journal.pone.0032397 (PMC3289653; doi:10.1371/journal.pone.0032397)
Supplement: Text S5 — Free energy for TAU hydrolysis in solution by steered-MD/PCVs. (DOC) [file pone.0032397.s014.doc]

# Text S5. Free energy for TAU hydrolysis in solution by steered-MD/PCVs

## Setup of cysteine methyl ester-TAU complex in solution

The cysteine methyl ester-TAU complex was built starting from the equilibrated CBAH-TAU Michaelis complex obtained from classical MD simulations. With exception of TAU substrate and Cys2, all the other residues composing the system were removed. The free carboxylate group of Cys was capped by O-methylation using Maestro software. Cysteine methyl ester (Cys-OMe) in complex with TAU was solvated using a box of TIP3P water molecules (Figure S4). The resulting system was equilibrated with 1.0 ns of MD in the NPT ensemble and periodic boundary conditions, using the sander module of AMBER10. To keep the geometry of the reactants close to the one of the CBAH-TAU Michaelis complex, a mean square deviation based harmonic restraints with a spring constant of 5 kcal/mol Å^-2^ were initially applied to C(O)NH of TAU atoms and S, Cβ, Cα, N_1_, H_1_ and Cys-OMe atoms.


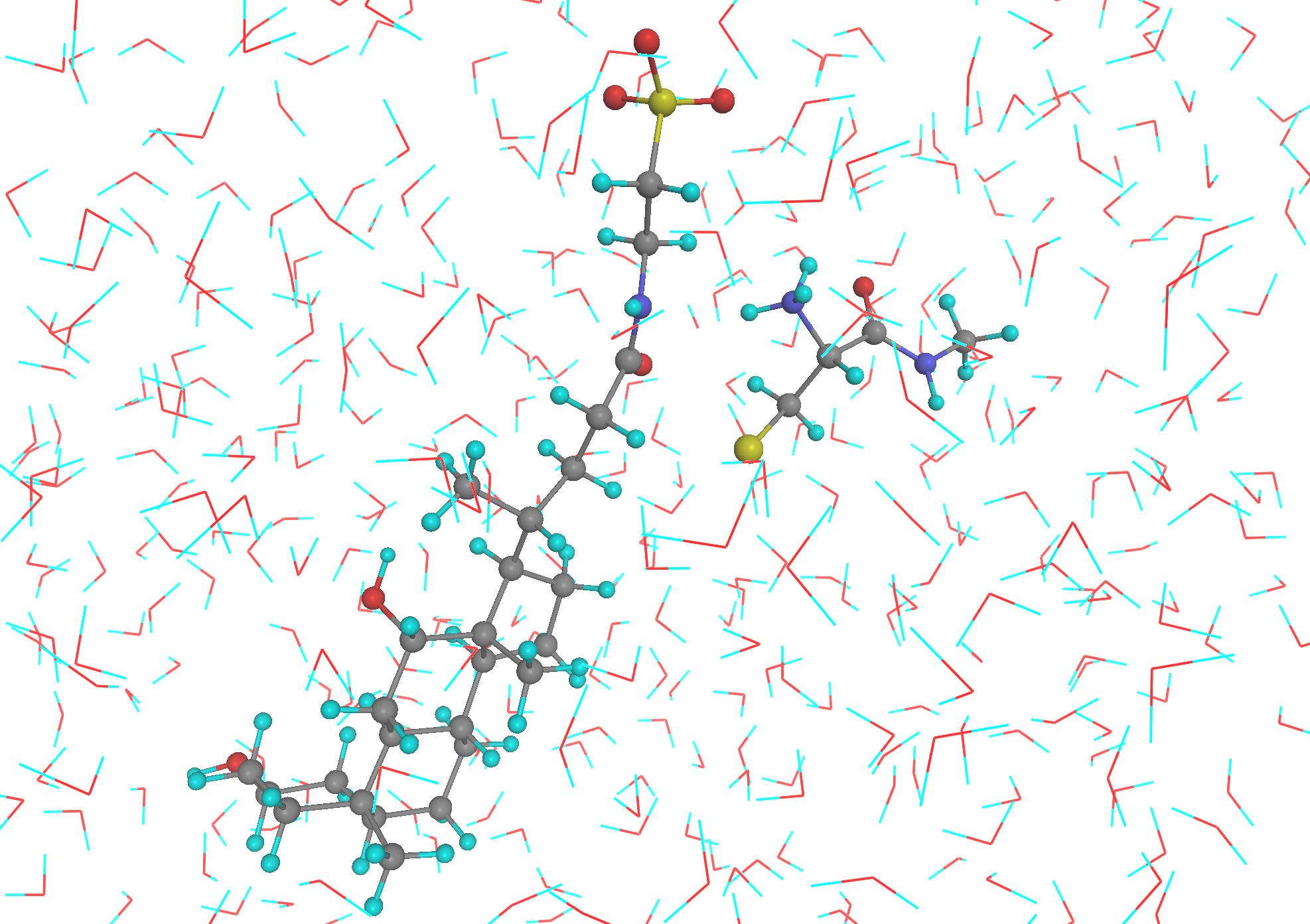


**Figure S4. Reactant complex formed by Cys-OMe and TAU substrate**. Reactant complex formed by Cys-OMe and TAU substrate as a model system to simulate amide hydrolysis in solution. Water molecules within 6 Å from the reactive center are displayed.

## QM/MM simulations

The equilibrated system was partitioned in two regions according to the QM/MM protocol employed for the full system (see main text). The Cys-OMe atoms and the 2-pentanamido ethansulfonic acid fragment of TAU were treated with the SCC-DFTB approach implemented in AMBER10, while all the other atoms (solvent molecules and a portion of TAU substrate) were treated in the MM framework using the AMBER99SB force field.

Also for the reaction between zwitterionic Cys-OMe and TAU, an initial guess path was generated by steered-MD, using simple reaction coordinates (RCs). TAU hydrolysis in aqueous solution was initially simulated considering three consecutive events (**A**, **B**, and **C** of Figure S5).

**
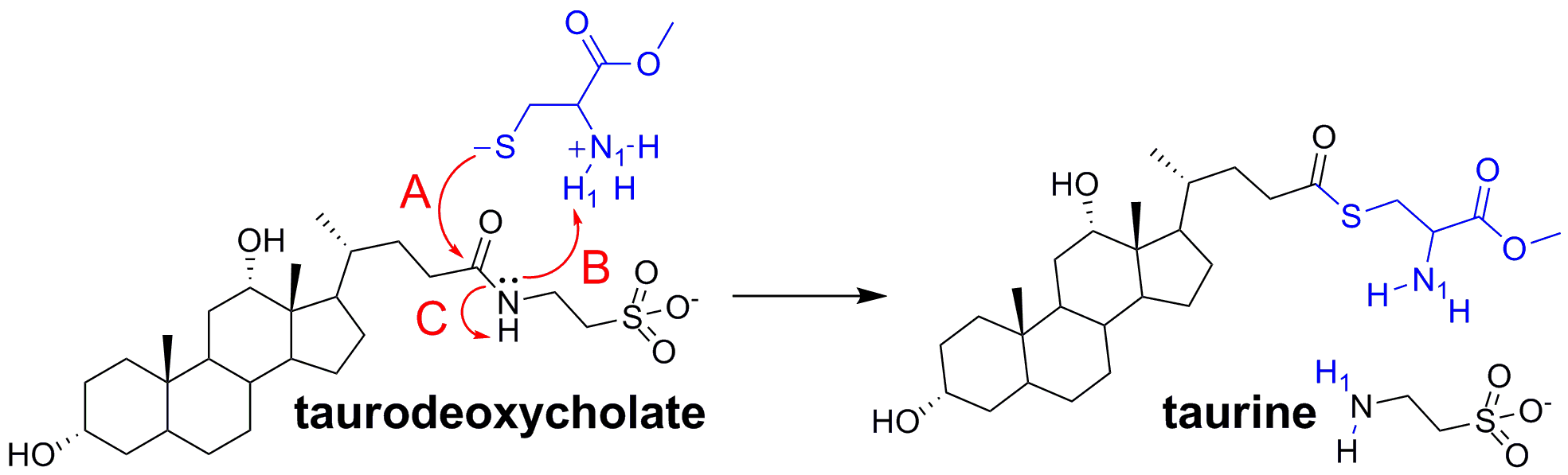
**

**Figure S5. First step of TAU hydrolysis by Cys-OMe in solution.** A, B, and C are key steps for the cleavage of TAU amide bond.

These RCs were pulled to their target values by means of steered-MD (force constant = 300 kcal/mol Å^-2^; overall simulation time = 10 ps). From the resulting trajectory, a set of 30 frames was extracted, and used for defining PCVs in steered-MD simulations. In PCVs representation the following atoms were considered: C(O)NH of TAU fragment, and S, Cβ, Cα, N_1_, H_1_ atoms of Cys-OME. From the initial steered-MD trajectory, 30 equally spaced frames were selected, with an average root mean square deviation of 0.11 Å. *S* was pulled to its target value by using a spring constant of 300 kcal/mol at a velocity of 0.5 *S* units per ps. *Z* was constrained by a quartic potential to allow a relative freedom of the system to relax, while preventing the system to escape the reactive region. The limit over *Z* was set to 0.005 Å^2^, with a force constant of 200 kcal/mol Å^-8^.

The activation barrier of TAU hydrolysis was estimated from the steered-MD work profile and it is reported in Figure S6, together with the work profile of the reaction catalyzed by CBAH and obtained via steered-MD.

**Figure S6.** **Comparison between TAU hydrolysis in CBAH and in solution.** Activation barriers for the first step of TAU hydrolysis catalyzed by CBAH (line) and in aqueous solution (dashed line), estimated from work profiles obtained from steered-MD/PCVs simulations. In the lower panel, relevant distances are plotted as a function of *S* (progress along the path).

At the transition state, the TAU-Cys adduct in water and the one in the enzyme showed a very similar geometry (Figure S7). Nevertheless, the additional free energy cost required for the reaction in water showed that the enzyme environment played a crucial role in promoting the proton transfer through pre-organized active site dipoles.


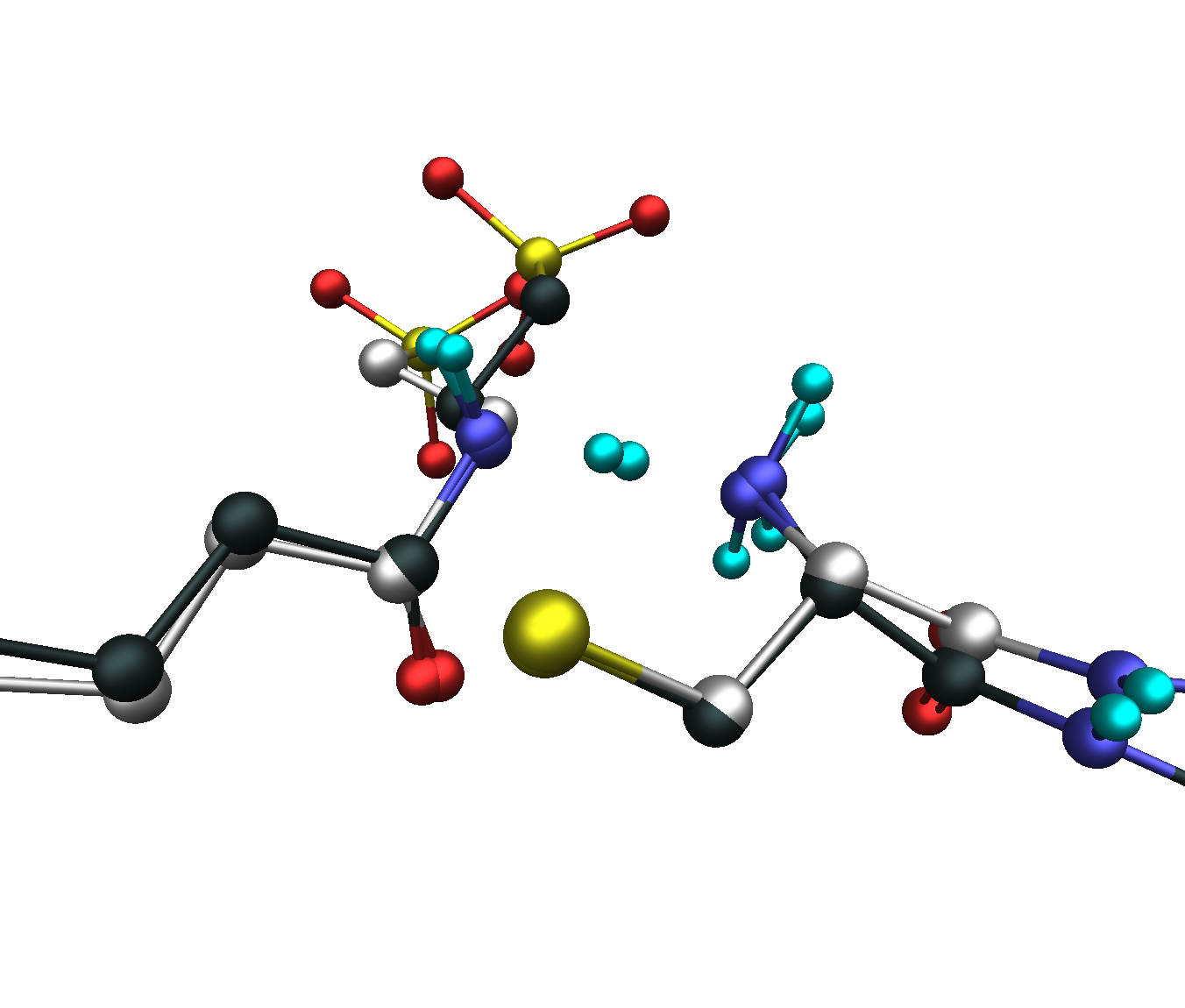


**Figure S7.** **Superposition of the transition state (TS) structures for the reaction in solution and in CBAH.** TS structures were identified along steered-MD/PCVs simulations. The TS geometry for the reaction in aqueous solution is reported with white carbon, while the TS geometry for the enzyme catalyzed reaction is reported with black carbons.
