# Supplementary material for: A Catalytic Mechanism for Cysteine N-Terminal Nucleophile Hydrolases, as Revealed by Free Energy Simulations
Source: PLoS One. 2012 Feb 28;7(2):e32397. doi: 10.1371/journal.pone.0032397 (PMC3289653; doi:10.1371/journal.pone.0032397)
Supplement: Text S6 — Zero point charge mutants of CBAH. (DOC) [file pone.0032397.s015.doc]

# Text S6. Zero point charge mutants of CBAH

On the basis of CBAH-TAU complex, several mutant models were built and employed for steered-MD/PCVs simulations. Specifically, single-mutant model of CBAH were built by zeroing the atomic charges of the desired residue, similarly to the procedure developed by Li and Cui

([[1]](#endnote-2)). In total, five zero-point charge CBAH mutants were built, i.e. Arg18, Asp21, Asn82, Asn175, Arg228, starting from the CBAH-TAU Michaelis complex obtained at end of the classical MD simulation.

Each mutant complex was thermalized via QM/MM MD for 20 ps at 300 K in NVT condition similarly to what performed for the wild type Michaelis complex (see main text). Then for each mutant, a steered-MD/PCVs run was carried employing the reaction path optimized for the CBAH-TAU wild type (composed by 30 frames) as a reference path. In all these steered-MD simulations the path variable *S* was steered from 1 (the reactants) to 30 (the products) applying a spring constant of 300 kcal/mol at a velocity of 0.5 *S* units per ps (for a total simulation time of ~50 ps).

The effect of the each mutant on the catalytic process (i.e. TAU hydrolysis) was estimated from the steered-MD work profile. Since dissipative work might affect the final energy values, the calculated barrier heights can be considered meaningful only for a comparison.

1. . Li G, Cui Q (2003) What is so special about Arg 55 in the catalysis of Cyclophilin A? Insights from hybrid QM/MM simulations. *J Am Chem Soc* 125: 15028-15038. [↑](#endnote-ref-2)
